# Household Readiness to Care for Mild and Asymptomatic COVID-19 Cases at Home, Southwest Ethiopia: A Community-based Cross-Sectional Study

**DOI:** 10.4314/ejhs.v32i6.3

**Published:** 2022-11

**Authors:** Lelisa Sena Dadi, Bekele Desse Kefeni, Hanan Kadi, Enku Kifle, Elias Ali Yesuf, Gelaw Hailemariam, Zerihun Kura Edosa, Tewedaj Befekadu

**Affiliations:** 1 Department of Epidemiology, Faculty of Public Health, Jimma University, Jimma, Ethiopia; 2 Jimma Zone Health Office, Jimma, Ethiopia; 3 Jimma University Medical Center, Jimma, Ethiopia; 4 Department of Health Policy and management, Faculty of Public Health, Jimma University, Ethiopia; 5 Department of Emergency medicine, Jimma University Medical Center, Jimma, Ethiopia; 6 Jimma Town Health Office, Jimma, Ethiopia

**Keywords:** Asymptomatic and Mild COVID-19 Cases, community readiness, household, Jimma Zone

## Abstract

**Background:**

Corona virus disease (COVID-19) continued with its notorious effects overwhelming health institutions. Thus, home-based identification and care for asymptomatic and mild cases of COVID-19 has been recommended. Therefore, the objective of this study was to assess the level of household readiness for caring asymptomatic and mild cases of COVID-19 at home.

**Methods:**

A community-based cross-sectional study was conducted from March-June 2021 on randomly selected 778 households. Data entry and analysis were carried out using EpiData and SPSS version 25, respectively. Multivariable logistic regression was modeled to identify independent predictors of community readiness.

**Results:**

Overall readiness of the community was very low (43.8%). Factors positively affecting household readiness were male household heads (AOR = 1.6; 95%CI: 1.05, 2.45), primary (AOR=2.0; CI:.62, 1.59) and higher (AOR = 1.90; 95%CI: 1.04, 3.45) educational level of the respondents, number of rooms within household (AOR = 1.22; CI: 1.03, 1.46), having additionally house (AOR = 2.61; CI: 1.35, 5.03), availability of single use eating utensils (AOR = 2.76; 95%CI: 1.66, 4.56), availability of community water supply (AOR = 8.21; 95% CI: 5.02, 13.43), and community participation and engagement (AOR = 2.81; 95% CI: 1.93, 4.08) in accessing transport, water and sanitation.

**Conclusions:**

The community was less prepared in terms of housing, infection prevention, water and sanitation. Considering alternative options including universal coverage of vaccine is important; designed behavioral change communications can enhance community participation and engagement in improving access to transport, water and sanitation to reduce risk of infections.

## Introduction

Corona virus disease of 2019 (COVID-19) pandemic has rapidly spread throughout the World since its occurrence in the late December 2019 in Wuhan, China (1). It has remained to be a top public health agenda (2). Ethiopia reported an index case of COVID-19 on March 13, 2020 (3). Since then, the notorious effects of the pandemic has continued (4) owing to inadequate adherence to prevention methods, low level of knowledge, and practices, poor socioeconomic status, and beliefs of the society (5, 6) resulting in hundreds of thousands cases, and loss of several thousand lives.

The pandemic has adversely affected several aspects of human lives including socioeconomic, culture, demographics, psychosocial, and health (7, 8). In particular, it has exerted its direct and notable pressure on health facilities such as increased patient costs, burdens on health care workers, competing for limited resources of health facilities (9–11). According to a systematic review, there was an overall 37% reduction of health services utilization in 20 countries due to the pandemic (12). A scoping review also highlighted that the pandemic resulted in several adverse maternal health consequences, including mental problems, increased domestic violence, decreased prenatal visits, and loss of jobs (13). Similar findings also reported from Ethiopia substantial reduction in reproductive, maternal, and newborn health care utilization, and significant shrinkage of both elective and emergency surgical volumes (14,15).

Moreover, the local (16–18) and global (19) trends of the disease show that the propagation of the pandemic has still continued at larger scale and interruption of the pandemic in near future is unlikely. Furthermore, the fact that more virulent and fast transmissible new variants of the virus have been emerging over time (20, 21), prompts that the pandemic requires additional options in order to withstand its mulifaceted impacts.

Consequently, caring for all cases at health facilities is recognized to be impractical. A study finding (22) has shown that even severe cases can be taken care of at patient homes. Home-based care for mild COVID-19 cases by family physicians has also been shown reduction of time gap for resolutions of major symptoms and rate of hospitalization (23). Thus, as a feasible option, identifying, and caring for mild and asymptomatic cases of COVID-19 at home with proper infection prevention and control has been recommended to reduce the health care burdens, and patient costs (24).

On the other hand, studies have implied the need for assessing challenges related to poor housing, overcrowding, inadequate water and sanitation (25) and understanding of the households' readiness in the already overcrowded home environment as important concerns. The need for assessing the readiness for home-based palliative care for COVID-19 cases has also been suggested (26). Therefore, the aim of this study was to assess household readiness for taking care of mild and asymptomatic COVID-19 cases at home in Jimma Zone, Southwest Ethiopia.

## Methods

**Study area, period, and design:** A community-based cross-sectional study was conducted from March-June 2021 in seven districts of Jimma zone, and four kebeles (smallest administrative units) of Jimma Town. Jimma zone consists of 20 districts and one special town (Agaro Town), 46 urban and 512 rural Kebeles. The zone has an estimated 3,518,260 population and 732,970 households. Jimma Town is a capital town of Jimma zone, which is located 355 km away from Addis Ababa to southwest. Jimma town administration has 17 kebeles, 41,535 households and 205,715 population. From the rural districts, Mencho, Nono-Benja, Kersa, Shebe-Sombo, Gumay, Omo-Nada, and Setema were selected. From Jimma town, Becho Bore, Mentina, Ginjo and Setosemero kebeles were included in to the study.

**Sampling procedure and sample size estimation:** Stratified cluster sampling methods was applied to reduce errors in cluster (urban and rural areas). A multi-stage sampling technique was used to select representative samples of independent study districts and kebeles in each stratum. After random selection of districts and kebeles, the sample was proportionally allocated to the size of the households in the respective kebeles. Similarly, simple random sampling procedure was applied to the selection of households. Sample size was estimated using single population proportion formula assuming maximum proportion, which is equal to 50% (P = 0.5) and taking 95% confidence level with ±5% precision, 10% non-respondent rate, expected 25–30% proportion among the cluster and the design effect of 1.8. Accordingly, 778 households (respondents) were included to the study.

**Measurements and operational definitions**: The dependent variable in this study is the community readiness for COVID-19 home-based care for mild and asymptomatic COVID-19 cases. It was indicated as ready if the respondents have good knowledge, increased vulnerability perception, positive attitude, and good practice towards prevention measures. Additionally, the participants were asked ten questions to assess “community readiness toward COVID-19 prevention. Each item has a “Yes” or “No” response giving a score of 1 and 0 (i.e., a score of 1 was given for “Yes”, and a score of 0 was given for “No”). The higher values indicate higher readiness to adapt to COVID-19 prevention. Depending on the mean score of readiness assessment questions, respondents who scored above the mean score were considered as having “Good readiness” and those who scored mean or below were considered as having “Poor readiness”. Likewise, the participants' readiness was coded as “1” for good readiness and ‘'0″ for Poor readiness.

**Data collection tool and quality assurance**: A questionnaire was prepared from review of related literature (27, 28). The questionnaire consists of socio-demographic characteristics of the respondents; housing, water and sanitation status; knowledge and practices of infection prevention and control of the heads of households.

The questionnaire was prepared in English. Then, it was translated to local language (Afan Oromo) and back translated to English by language experts to maintain consistency. Data collection tool was pre-tested in non-selected kebeles to ensure its appropriateness for the data collection. Data collectors and supervisors were trained on content of the questionnaire, interview techniques, and ethics of research. Supervisors strictly monitored the data collection process, and any missed or incorrectly recorded data were checked every day and corrective actions were taken immediately.

**Statistical analysis**: Data entry and analysis were done using EpiData and SPSS version 25, respectively. Descriptive statistics were done to summarize data. Then, bivariate was carried out to determine the variables to be included in the multivariable model based on P value ≤ 0.2. Finally, multivariable logistic regression was done to identify independent predictors of community readiness and to control confounding variables among independent variables. Adjusted odds ratio and confidence interval (CI) were respectively used to measure statistical associations and their statistical significances.

**Ethics and consent**: Ethical clearance was obtained from the Ethical Review Committee of Jimma University Institute of Health. Support letters were also obtained from Jimma Emergence Operation Center (JEOC), Jimma Zonal Health Office and Jimma Town Health Office and communicated to the respective districts and kebele administration of the study areas. Informed consents were obtained from all heads of the study households who participate in the interviews. Individual information is kept anonymously and the findings are communicated in aggregated forms. Materials and equipment used for this study were handled and disposed safely considering environmental safety.

## Results

**Socio-demographic characteristics of the study population:** Total of 778 (100% response rate) respondents were included into the study. More than two-third (68.9%) of the respondents were males. A quarter (25.4%) and one-third (33.5%) of them were within the age range of 25–34 and 35–45 years, respectively. Majority of the respondents were married (86.1%), Muslims (88.8%) and more than three-fourth (76.3%) of them were rural residents while about two-third of them were illiterate (67.4%) and farmers (69.8%). There were also 4562 male (49.52%) and females (50.48%) family members of the respondents. Less than 18, 18–64, and ≥ 65 years individuals accounted for 1599 (35.05%), 2841(62.28%), and 122 (2.47%), respectively. The household size was closely six individuals (mean ± SD = 5.86 ± 2.58) (Table 1).

**Table 1:** Socio-demographic characteristics of the study participants, Jimma Zone, Southwest Ethiopia, March-June 2021

Variables	Categories	Number (%)
Sex of respondents	Male	536 (68.9)
	Female	242 (31.1)
Age of respondents	< 25 years	65(8.4)
	25–34	198 (25.4)
	35–44	261(33.5)
	45–54	140 (18.0)
	≥ 55	114 (14.7)
Marital status of respondent	Single	69(8.9)
	Married	670(86.1)
	Divorced/Widowed	39 (5.0)
Residence of respondent	Urban	184(23.7)
	Rural	594(76.3)
Respondent highest level of education	Illiterate	524(67.4)
	Primary	168(21.6)
	Secondary or above	86(11.1)
Respondent religion	Muslims	691(88.8)
	Christians	87 (11.2)
Household livelihood of respondent	Farmer	543 (69.8)
	Merchant	100 (12.9)
	Government employee	67 (8.6)
	Others	68 (8.7)
Family members by sex	Male	2259 (49.52)
	Female	2303 (50.48)
Family members by age group	<18 years	1599 (35.05)
	18–64 years	2841(62.28)
	> 64 years	122 (2.47)
HH size by sex	Male, mean (Std. D)	2.90 (1.67)
	Female, mean (Std. D)	2.96 (1.59)
	Total, mean (Std. D)	5.86 (2.58)

**Housing condition**: Most (89.5%) of the respondents have their own houses. Only below half (48.6%) of the houses had electricity and about a quarter (26.3%) had television. The main materials of the walls were wood/mad (64.7%), and bricks (29.7%). Most of the houses had earthen floor (88.9%), and corrugated iron sheets (96.9%) roofs. The main source of energy for most (88.9%) of the households were woods. Most (90%) of the households did not have additional houses. The total rooms of the houses were 2567 implying an average 3.30 (±1.21) individuals per a living room. There were also total of 1823 doors and 1903 windows, implying 2.34 (±1.01) doors and 2.45 (±1.04) widows per a house, respectively.

Only 133 (17.1%) households had rooms that had been designed fully with doors and windows separately. More than half (56.8%) of households had some rooms that were partially designed with separate doors and windows while more than a quarter of them (26.1%) did not have such arrangements at all. Similarly, only 108 (13.9%) of the households had rooms with doors that direct to outside while more than half (58.5%) of them reported that only some of the rooms had doors that direct to outside. Sill, more than a quarter (27.6%) of the households did not have doors that direct to outside. Only half (50.1%) of the available widows were reported to be openable throughout the day time (68.2%), and half of the day time (24.1%).

Only four in ten (40.5%) of the households had ventilated separate rooms for COVID-19 suspected cases. Only close to a quarter of the respondents reported that a person with suspected COVID-19 can have the chance to be provided single use eating utensils (Table 2).

**Table 2:** Housing Status of the study participants, Jimma Zone, Southwest Ethiopia, March-June 2021

Variables	Categories	N (%)/Mean (Std. D)
Electricity	Yes	378 (48.6)
	No	400 (51.4)
Television	Yes	205 (26.3)
	No	573 (73.7)
Main material of the floor	Earth	692 (88.9)
	Cement	77 (9.9)
	Brick	9 (1.2)
Main material of the roof	Corrugated iron sheet	754 (96.9)
	Thatched	24 (3.1)
Main source of fuel	wood	692 (88.9)
	electric	86 (11.1)
Ownership of houses	Owner	696 (89.5)
	Rental	82 (10.5)
Sharing Utilities within households	Yes	152(19.5)
	No	626(80.5)
Additional houses	Yes	78 (10.0)
	No	700 (90.0)
Ventilation and access of the houses	Mean # of rooms per house (Std. D)	3.30 (±1.21)
	Mean # of doors (±Std. D)	1823; 2.34 (±1.01)
	Mean# of windows (±Std. D)	2.45 (±1.04)
Rooms designed with doors and windows	Yes, all the rooms	133 (17.1)
	Yes, some of the rooms	442 (56.8)
	No	203 (26.1)
Each room directs to outside separately	Yes, all the rooms	108 (13.9)
	Yes, some of the rooms	455 (58.5)
	No	215 (27.6)
well-ventilated single room	yes	315 (40.5)
	No	463 (59.5)
Waste bag in room is used for tissues, facemasks, and other	Yes	271 (34.8)
	No	507 (65.2)
Share spaces with non-case members	Yes	152 (19.5)
	No	626 (80.5)
Spaces and utilities are shared	Bedroom	96 (62.7)
	Saloon	50 (32.7)
	Rest room	7 (4.6)
Window are openable	Yes	390 (50.1)
	No	388 (49.9)
If windows are openable:	Throughout the day	266 (68.2)
	For half of the day	94 (24.1)
	Unknown	30 (7.7)
Single use of eating utensils	Yes	194 (24.9)
	No	584 (75.1)
Total		778 (100)

**Infection prevention and control**: Just above a quarter (26.9%) of the study participants reported availability of a designated person to care of suspected COVID-19 cases within their households. Again, only 39.2% of them reported that they get personal protective equipment (PPE) from authorized health institutions. Still, only one-third (33.7%) of them reported use of gloves and protective clothes for cleaning surfaces and small proportion of the respondents reported that touchable surfaces are frequently cleaned, and 41.4% of the respondents used proper detergents for cleaning.

About half (53.7%) of the respondents reported that they use PPE while cleaning rooms. More than half (56.0%) of them use soap or detergents for cleaning utensils, 25.8% and 68.0% of them for cleaning bathrooms and surfaces, respectively. The respondents reported that they would seek medical care in case of suspecting COVID-19 from public health facilities (55.3%), COVID-19 testing center (32.8%), and private health facilities (12.0%) (Table 3).

**Table 3:** Infection prevention and control practices of the study participants, Jimma Zone, Southwest Ethiopia, March-June 2021

Variables	Responses	Number (%)
Was there suspected of COVID-19 symptoms in the family members	Yes	95 (12.2)
	No	683 (87.8)
Assigned/ready to assign a care giver for suspected COVID-19 cases	Yes	209 (26.9)
	No	569 (73.1)
Care giver get/purchase the PPE from	Authorized health	305 (39.2)
	Any open markets	473 (60.8)
Use gloves and protective clothing when cleaning surfaces	Yes	262 (33.7)
	No	516 (66.3)
Frequently touchable surface of the room cleaned	Yes	305 (39.2)
	No	473 (60.8)
Have contamination frequently with proper detergent	Yes	322 (41.4)
	No	456 (58.6)
Cleaning rooms using PPE	Yes	418 (53.7)
	No	360 (46.3)
During homecare placed into a waste bin with a lid	Yes	320 (41.1)
	No	458 (58.9)
Use soap or detergent for cleaning of utensils	Yes	436 (56.0)
	No	342 (44.0)
Utilized bathroom after cleaning surfaces	Yes	201 (25.8)
	No	577 (74.2)
use glove and protective clothing when cleaning surfaces	Yes	529 (68.0)
	No	249 (32.0)
Where to seek medical support whenever for suspect COVID-19	COVID-19 testing centers	255 (32.8)
	Public health facilities	430 (55.3)
	Private health facilities	93 (12.0)

**Factors affecting readiness of households:** At bivariate analysis, variables such as age, residence, family size, marital status, number of doors within a house, presence of suspected individuals within a household, having separate rooms with doors, and windows that direct to outside, sharing spaces, and utensils, and presence of health institutions within distance of five kilometers were associated with readiness of caring for mild and asymptomatic cases of COVID-19 at home at P value ≤ 0.2. However, the associations of those variables were insignificant when treated by multivariable logistic regression at P value ≤ 0.05.

On the other hand, sex of head of household, educational status of the respondents, number of rooms within household, having additional living house, availability of single use eating utensils, community water supply, community participation and engagement in road maintenance were found to be significantly associated with readiness of care at home both at bivariate and multivariable logistic regression analyses. Household with male heads were more (AOR = 1.6; 95%CI: 1.05, 2.45) likely to be ready for caring of the cases at home compared to female headed households. Households where the respondents had education were about two times (AOR = 1.90; 95%CI: 1.04, 3.45) more likely to get ready for caring the cases at home compared to households whose respondents were illiterate.

Households with additional living house were two times more (AOR = 2.61; 95%CI: 1.35, 5.03) likely to be ready for caring the cases at home compared to those who haven't additional house. Households who have single use eating utensils were about three times (AOR = 2.76; 95%CI: 1.66, 4.56) more likely to be ready for caring the cases at home compared to households who did not have them. Households who have community water supply at recommended distance from the household were eight times (AOR = 8.21; 95% CI: 5.02, 13.43) more likely to be ready for caring the cases at home compared to households who live where there was no community water supply. Households who live where community participate and engage in activities such as road maintenance were more (AOR = 2.81; 95% CI: 1.93, 4.08) likely to get ready for caring the cases at home compared to their counterparts (Table 4).

**Table 4:** Factors affecting community readiness for caring mild and asymptomatic cases of COVID-19 at home, Jimma Zone, Southwest Ethiopia, March-June 2021

Variables	Categories	Readiness		P-value (COR)	COR (95%CI)	AOR (95%CI)
		Yes	No			
Sex of Household head	Male	257	279	0.001	1.73(1.27, 2.37)	1.61(1.05, 2.45) *
	Female	84	158		1	1
Age	Mean (± SD)	39.37(12.56)		0.037	1.01(1.001, 1.02,)	
Residence	Urban	76	124	0.054	1.38(0.99, 1.92)	
	Rural	265	313		1	
Educational status	Illiterate	209	315		1	1
	Primary	84	84	0.021	1.51(1.06, 2.14)	1.996(.62, 1.59)
	Higher	48	38	0.006	1.90(1.20, 3.02)	1.90(1.04, 3.45) *
Family size	Mean (± SD)	5.86(2.584)		0.001	1.10(1.04, 1.17)	
Marital status	Single	20	49		1	
	Married	303	367	0.011	2.02(1.18, 3.48)	
	Divorced	8	11	0.280	1.78(0.62,5.09)	
	Widowed	10	10	0.085	2.450(.884,6.789)	
Number of rooms in HH	Mean (± SD)	3.30(1.21)		0.003	1.20(1.07, 1.36)	1.22(1.03, 1.46) *
Number of doors in HH	Mean (± SD)	2.34(1.013)		0.001	1.29(1.11, 1.49)	
additional living house	Yes	56	21	0.000	3.91(2.31, 6.60)	2.61(1.35, 5.03) *
	No	284	416		1	1
Persons with COVID-19 symptoms	Yes	106	36 .000	5.02 (3.33, 7.58)		
	No	235	401		1	
Room designed with doors and windows separately	Yes, all	69	64	.048	1.56 (1.003,2.42)	
	Yes, some	189	253	.655	1.08(0.77,1.51)	
	No	83	120		1	
Separate door for each room	Yes, all	55	53	.144	1.41 (0.89, 2.25)	
	Yes, some	195	260	.897	1.02(0.74, 1.42)	
	No	91	128		1	
Sharing utilities with other households	Yes	106	46		1	
	No	235	391	0.000	3.83(2.62,5.62)	
Shared spaces with others	Yes	106	46		1	
	No	235	391	0.000	3.83 (2.62, 5.62)	
Single use eating utensils availability	Yes	139	55	0.000	4.78(3.35, 6.82)	2.76(1.66, 4.56) *
	No	202	382		1	1
Community water supply availability	Yes	306	263	0.000	5.78(3.88, 8.62)	8.21(5.02,13.43) *
	No	35	174		1	1
Health facility ≤ 5km	Yes	285	348	0.162	1.30(0.90,1.88)	
	No	56	89		1	
Community participation and engagement	yes	231	110	0.000	2.36(1.75, 3.16)	2.81(1.93, 4.08) *
	No	206	231			1

## Discussion

This study has assessed the level of community readiness for caring mild and asymptomatic cases of COVID-19 at home from accessibility, acceptability, and community engagement view points. The overall readiness of the community was found to be very low (43.8%). The studied households had larger family size than the national average (5.86 vs 4.7) (29). However, it is in agreement with previous local findings (30–32), which highlight that more than half of Ethiopians live in overcrowded housing units. Similarly, a study, based on DHS of 54 African countries, reported that at least 50% of the African population live in larger households with six or more individuals was estimated to be 56.4%, more than two-third (71%) lack on-site water supply, and significant proportion (45%) of the households share toilets (33). Such crowded home settings implies high possibility of disease transmission among member of the households.

In this study, majority (88.9%) of the floors of the houses were wood/earth, and only a quarter (25.4%) of them had utilities within the houses, implying non-suitability of the houses for cleaning and disinfections. The EDHS-2019, also reported that 70% of floors of the Ethiopian houses are earth or sand, and 10 % are dang (29). Moreover, only small proportions of the houses had additional houses for isolation (10.0%), doors and windows designed to separately direct to outsides (17.1%), ventilated single rooms for COVID-19 suspected cases (40.5%), dust bins for wastes (34.8%), can provide single use utensils for suspected cases (24.9%), and assigned or ready to assign (26.9%) persons to care of COVID-19 suspected cases.

A study conducted in Gondar Town, northern Ethiopia also reported similar findings that water scarcity, poor sanitation, and hygiene practices account for 42.9%, 67%, and 51.7%, respectively (34). In general, such findings highlight compromised housing standards in many Ethiopian settings, including the present study setting. These conditions make the feasibility of safe home-based care for infected cases impractical.

Regarding to the role of housing and living conditions in prevention and control of the pandemic, different findings (3,35–38) confirmed that the risk of COVID-19 infection increases in a community where basic home facilities are lacking. Poor housing are strongly associated with both increased risk of COVID-19 infections and mortality. Nevertheless, the studied household were found to be less prepared in this aspect as well. Only 39.2% of the respondents reported to have possibility of purchasing sanitizers from authorized health institutions. Again, only a third (33.7%) of the respondents reported that they use gloves and protective clothing when cleaning the surfaces.

About four in ten (41.4%) of them reported that contaminations are cleaned frequently with proper detergent and the same proportion of them had dust bin with lid for wastes. Just above half (53.7%) of the households use PPE when cleaning rooms. Similar proportion (56%) of them use soaps or other detergents for cleaning utensils and only a quarter (25.8%) of them utilize bathroom after cleaning surfaces. These findings seem better than reports of EDHS 2016 (39) where soap and water were observed only in 28.0% urban and 7.0% rural households. Similarly, it has been reported that, across Africa, more than half of the people lack observed soap/washing facilities in their households (33).

Moreover, studies (6,40) also highlighted that non-pharmaceutical public health measures are not practical in Ethiopian contexts due to beliefs, low perceived risks of the disease, inadequate knowledge, and attitudes. Thus, from infection prevention point of view, the available evidences do not show the feasibility of caring for asymptomatic and mild cases of COVID-19 at home since the disease can easily transmitted in the absence of adequate PPEs and sanitary supplies.

Households with male educated heads, having additional living houses, and having single use eating utensils were positively and significantly associated with readiness of the households for taking care of mild and asymptomatic cases of COVID-19 at home. This is a reflection of socioeconomic status where the better offs are better prepared for and ready to care for the +cases at home. Earlier studies have already reflected that in socioeconomically poor setting, people have low readiness, low knowledge, and poor practices of responses to the pandemic (33, 35, 36). Similarly, households who live where community water supply is available and where the community participate and engage in accessing roads were more ready. These findings are similar with findings of a scope review (41) in some aspects that higher education, and availability of community services were significantly associated with increased home care intensity.

There can be social desirability bias from the side of study participants in responding to some of the questions. Although pre-test has been made, the tool has not been validated locally. Due to the limited scope/nature of quantitative survey, the study might not fully captured the inside view of the respondents.

In conclusion, the community was less prepared to take care of mild and asymptomatic cases of COVID-19 at home in terms of infection prevention and control, and physical structure of the houses. Soaps and detergents were scarcely available in most households or not at all. Most houses are overcrowded, unsuitable for cleaning, and inadequately ventilated. Therefore, alternative options such as universal coverage of vaccine for high-risk individuals need to be considered. A designed behavioral change communications can also enhance community participation and engagement in improving access to transport, water and sanitation as well as in reducing risk of infections.
